# Sexual Function and Quality of Life in Brazilian Transgender Women Following Gender-Affirming Surgery: A Cross-Sectional Study

**DOI:** 10.3390/ijerph192315773

**Published:** 2022-11-27

**Authors:** Lísia Maya Monteiro Petry Jardim, Taís Marques Cerentini, Maria Inês Rodrigues Lobato, Ângelo Brandelli Costa, Dhiordan Cardoso da Silva, Karine Schwarz, Anna Martha Vaitses Fontanari, Maiko Abel Schneider, Tiago Elias Rosito, Valentina Lucia La Rosa, Elena Commodari, Patrícia Viana da Rosa

**Affiliations:** 1 Department of Physiotherapy, Federal University of Health Sciences of Porto Alegre, Porto Alegre 90050170, Brazil; 2 Post Graduation Program in Sciences of Rehabilitation, Federal University of Health Sciences of Porto Alegre, Porto Alegre 90050170, Brazil; 3 Gender Identity Program, Department of Psychiatry, Hospital de Clínicas de Porto Alegre, Porto Alegre 90050170, Brazil; 4 Department of Psychology, Pontifical Catholic University of Rio Grande do Sul, Porto Alegre 90050170, Brazil; 5 Mood Disorder Program, Department of Psychiatry and Behavior Neuroscience, Youth Wellness Centre, McMaster University, Hamilton, ON L8S 4L8, Canada; 6 Gender Identity Program, Department of Urology, Hospital de Clínicas de Porto Alegre, Porto Alegre 90050170, Brazil; 7 Department of Educational Sciences, University of Catania, 95124 Catania, Italy

**Keywords:** male-to-female transgender, surgery, sexual health, quality of life, urinary function

## Abstract

This cross-sectional and descriptive study aimed to evaluate the sexual function, urinary function, and quality of life of 26 Brazilian trans women who have undergone gender-affirming surgery (GAS) using the gold standard technique (penile inversion vaginoplasty) in the Gender Identity Transdisciplinary Program at Hospital de Clínicas de Porto Alegre, Brazil, between March 2016 and July 2017. The Female Sexual Function Index, the SF-36 Health Survey, and the International Consultation on Incontinence Questionnaire-Short Form were used. Regarding their surgical results, 84.6% of the women said they were satisfied, 73.1% were sexually functional, and 15.4% reported urinary incontinence not associated with surgery. Participants also reported a good quality of life, despite low scores of pain and physical vitality. Transgender women in our sample reported a good quality of life and sexual function after GAS. Further studies are required to improve the psychosexual wellbeing of this specific population.

## 1. Introduction

Gender dysphoria (GD) is a condition that affects people whose gender identity differs from their assigned sex at birth [1] and can cause intense psychological distress. In such cases, gender-affirming surgery (GAS) can be performed to modify the patient’s phenotypic sexual characteristics. However, it is a radical surgery that completely changes the anatomy of the genitalia and pelvic floor muscles, which can lead to complications that favor the development of sexual dysfunction (SD) and alterations in urinary function, both with a negative impact on the quality of life of transgender women [2]. A recent systematic review by Oles et al. [3] showed high patient satisfaction with genital procedures but little agreement between study methods. The authors emphasize that it is essential to standardize assessment instruments and methods through patient-inclusive and multidisciplinary consensus efforts. Another systematic review by Javier et al. [4] also showed surgical satisfaction and improved quality of life after surgery, although most studies have small samples and do not allow causal conclusions.

The most common complication after a vaginoplasty is vaginal stenosis, although fistulas, neovaginal prolapse, urinary incontinence (UI), and urethral stenosis can also occur [5]. As a result of these complications, transgender women may have difficulty having vaginal sexual intercourse with their partners [6]. Furthermore, UI can cause social restrictions and urethral stenosis resulting from shortening, and repositioning of the urethra can lead to urinary retention [2].

The pelvic floor can also suffer changes in muscle tone due to urine being held for long periods, which is a consequence of the prejudice that transgender people suffer when going to public bathrooms and hormonal treatment with estrogen and androgens, which can also lead to UI [2]. In addition, transgender women can develop SD, as it is affected by physical, psychological, and sociocultural factors [7,8].

Taking into account the anatomical and physiological changes resulting from GAS, it is necessary to understand the impact this treatment has on the health of transgender women. Therefore, this study aimed to describe and evaluate the sexual function, quality of life, and possible alterations in urinary function in transgender women who have undergone GAS in a hospital in Brazil.

## 2. Materials and Methods

### 2.1. Study Design

A cross-sectional and descriptive study using convenience non-probability sampling was conducted between March 2016 and July 2017 at the Hospital de Clínicas de Porto Alegre (HCPA) with transgender women who had undergone GAS. The study was approved by the Ethics Committee of the HCPA and the Universidade Federal de Ciências da Sade de Porto Alegre (UFCSPA) under registration number 51763815.7.3001.5345. The study was designed following the Declaration of Helsinki and the Committee on Publication Ethics (COPE) guidelines. In addition, the project, analysis, data interpretation, redaction, and revision followed Strengthening the Reporting of Observational Studies in Epidemiology (STROBE) guidelines to report observational studies, available at the EQUATOR (Enhancing the Quality and Transparency of Health Research) network [9] (see Table S1 in Supplementary Materials).

The sample was composed of transgender women who had undergone GAS at HCPA between 2002 and 2017, with or without active sexual life, as participants in the Gender Identity Transdisciplinary Program (PROTIG). Transgender women who had undergone GAS outside of HCPA (PROTIG), smokers who consumed more than three packs a day, people who used industrial silicone, and transgender men were excluded from the study. Smokers who consumed more than three packs a day were excluded because they were at increased risk of scar complications which could directly interfere with the results of this study.

Transgender women were invited to participate in the research in two ways: (a) they were present in the hospital (for urological and psychiatric follow-up consultations or participating in preoperative support groups) on the day the researcher collected data and (b) after a profile analysis of 160 people registered in the HCPA database, 50 transgender women were contacted by phone. Preference was given to mobile prefixes in Porto Alegre and its metropolitan area to facilitate adherence to the research.

All individuals had undergone GAS using the gold standard technique for transgender women (penile inversion vaginoplasty). This technique consists of inversion of the penile skin to construct the neovagina. The same experienced surgical team performed all surgeries after these patients met the technical and diagnostic criteria defined by the MS 2803 ordinance [10].

### 2.2. Gender Identity Transdisciplinary Program (PROTIG)

In Brazil, gender transition through the Unified Health System (UHS) was implemented by ordinance n° 170/GM/MS on 18 August 2008 [11] and by ordinance no. 457/SAS/MS on 19 August 2008 [12]. These ordinances allowed university hospitals to perform procedures such as GAS. Law No. 2803 [10], which redefined service protocols and extended the transition process to ambulatory care through SUS, was published on 19 November 2013. Approximately five university hospitals in the country perform this surgery. Among the most experienced, the HCPA has provided free and public assistance to transgender men and women since 1998 through PROTIG [2]. This program offers psychiatric, psychological, urological, gynecological, and social support, surgical treatment when necessary, and clinical support for people diagnosed with GD. According to Brazilian rules, to undergo this surgery, people must undergo multidisciplinary surveillance for at least two years, be over 21 years of age (a federal requirement for this specific medical procedure), have a positive psychiatric or psychological history, and be diagnosed with ‘gender incongruence of adolescence and adulthood’ according to the classification of the 11th revision of the International Statistical Classification of Diseases and Related Health Problems (ICD-11) [13].

### 2.3. Variables

A specific questionnaire was used to assess the sociodemographic profile: date of interview, medical record, age, weight, height, type of postoperative care, hormonal replacement therapy, associated diseases, tobacco/day, postoperative smoking, urinary changes, surgery site, date of surgery, number of surgeries, satisfaction with surgery outcomes, if the patient had or did not have an active sexual life, if the patient had or did not have sexual activity in the past four weeks, and if the neovagina was penetrated.

Participants responded to three other questionnaires: the Female Sexual Function Index (FSFI, to verify the prevalence of SD [14]), the SF-36 Health Survey (SF-36) to evaluate the quality of life [15], and the International Consultation on Incontinence Questionnaire (ICIQ-SF), which quickly assesses the impact of UI on quality of life and classifies urinary losses [16].

#### 2.3.1. Female Sexual Function Index (FSFI)

The FSFI is a tool used worldwide, created in 2000, to evaluate female sexual function through a multidimensional self-report [17] translated and validated in Portuguese [18]. It comprises 19 items that assess sexual function in the last four weeks with scores in six domains: desire, arousal, lubrication, orgasm, satisfaction, and pain or discomfort. The items are scored on a five-point scale, with low scores indicating lower levels of sexual function. The scoring is reversed only for items related to pain. If the score in any domain is equal to zero, the person did not report sexual activity in the last four weeks. For individual domain scores, the sum of each domain is first multiplied by a domain factor ratio to place all the domain totals on a more comparable scale and then subsequently summed to derive a total FSFI score. The six domain scores are subsequently summed to obtain the full-scale score. The final score can range from 2 to 36, and high scores indicate a better level of sexual function [14]. The FSFI is a valid instrument and is considered effective in diagnosing female SD. The cut-off score was 26.55, with scores lower than this indicating dysfunction. The authors also defined cut-off scores for each domain: desire, 4.28; arousal, 5.08; lubrication, 5.45; orgasm, 5.05; satisfaction, 5.04; pain, 5.51 [17,18].

#### 2.3.2. International Consultation on Incontinence Questionnaire (ICIQ-SF)

The ICIQ-SF simply and objectively evaluates the impacts of UI on quality of life and classifies urinary losses in both sexes [16]. It comprises four questions: three related to frequency, amount of leakage, and overall impact of UI on quality of life, and one self-diagnostic item to evaluate the causes or situations in which urinary loss occurs on a scale of eight items. The ICIQ Score (ICIQ E) is calculated by adding the values from questions 1, 2, and 3 and varies from 0 to 21. The impact on quality of life is defined according to the score of Question 3: (0) none, (1–3) mild, (4–6) moderate, (7–9) severe, and (10) very severe [19].

#### 2.3.3. SF-36 Health Survey (SF-36)

The SF-36 questionnaire comprises 36 items that evaluate eight domains: physical functioning, role—physical, bodily pain, general health, vitality, social functioning, role—emotional, and mental health. The scoring scale ranges from 0 to 100, where zero corresponds to the worst general health condition and 100 to the best general health condition [15].

### 2.4. Statistical Analysis

An independent investigator, without prior knowledge of the groups, performed all the statistical analyses. The Statistical Package for the Social Sciences (SPSS) 25.0 (Armonk, NY: IBM Corp.) for Windows was used to analyze the data. The Shapiro–Wilk test was used to verify the normality of the quantitative variables. Continuous variables with normal distribution were described as mean and standard deviation or median and interquartile ranges when asymmetric. Absolute and relative frequencies explained the categorical variables. Comparisons between the scores of the sexually active group in the last four weeks and those of the sexually inactive group in the previous four weeks in the Female Sexual Function Index were made using the Wilcoxon–Mann–Whitney test and associations between UI and variables overweight, postoperative care, hormone replacement, associated diseases, and smoking were found using the chi-square test with a statistical significance level of 0.05.

## 3. Results

**Figure 1:**
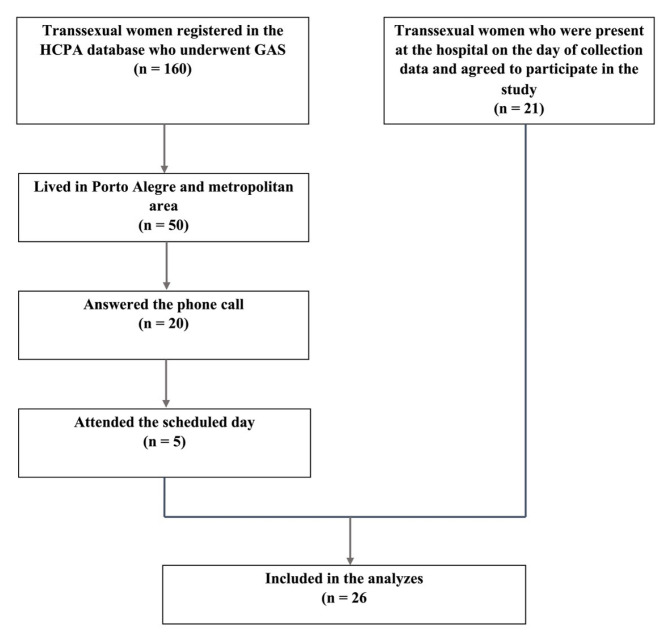
Flow chart of patient enrollment. HCPA: Hospital de Clinicas de Porto Alegre; GAS: Gender-Affirming Surgery.

Of the 26 transgender women who accepted to participate in the study, 21 were in the hospital when the data was collected. Five were contacted by phone and attended the agreed date. These data are represented in the flow chart reported in Figure 1.

In the final sample, age ranged from 23 to 64 years, and the mean body mass index was 25.26 ± 3.62. Approximately 30% of these women were smokers, 26.9% had associated diseases, and 26.92% had undergone aesthetic touch-ups in rectovaginal fistulas, vaginal, and urethral structures to repair complications. The other clinical and sociodemographic data are shown in Table 1.

Regarding their surgical results, 84.6% of the women said they were satisfied. Low depth/dilation was reported by 7.7% as one of the leading causes of dissatisfaction.

Of the transgender women interviewed, 80.8% reported having an active sexual life, and 73.1% reported having sexual intercourse in the last four weeks. However, 23.1% said that they did not have neovaginal penetration for the following reasons: absence of desire for penetration (15.4%), having little or no vaginal depth (3.8%), not having vaginal dilation (3.8%), and having rectovaginal fistulas (3.8%).

Approximately 34.6% of the sample reported urinary changes, and 19.2% reported difficulty emptying the bladder, having to force urine out, and feeling a burning sensation when urinating.

### 3.1. Sexual Function

The cut-off score for having or not having SD was 26.55 [17]. The mean score of the sample was 21.58 (Table 2), which qualifies the sample as sexually dysfunctional according to the general pattern.

However, when analyzing only the population that was sexually active in the last four weeks, the mean score was 26.67, which indicates that they are sexually functional. The domain with the lowest mean score was lubrication, and the highest was satisfaction.

Analyzing the results of the participants who were not sexually active in the last four weeks, the total score was 5.63. Although considered sexually dysfunctional, this group had a mean score greater than zero in the domains of desire and satisfaction.

### 3.2. Urinary Incontinence

As shown in Table 3, of the 26 transgender women interviewed, 15.4% reported UI. Among them, 3.8% had severe UI, 7.7% had moderate UI, and 3.8% had mild UI. Participants also reported urine losses before going to the bathroom (3.8%), losses without apparent reason (3.8%), losses during sleep (3.8%), and constant losses (3.8%). It is essential to note that all transgender women who complained of loss of urine said it had started before GAS.

In the chi-square test, we did not observe an association between UI and overweight variables (*p* = 0.188), postoperative care (*p* = 1.000), hormone replacement (no statistics were calculated because the variable was constant), and associated diseases (0.302). Only smoking was significant (*p* = 0.005).

### 3.3. Quality of Life

The quality of life standard can be observed in Table 4. The mean values for each questionnaire component were between 72.88 and 93.65. The lowest mean values were recorded in physical pain and vitality domains, while the highest was recorded in functional capacities and emotional aspects. In general, the sample had good health conditions.

## 4. Discussion

This study aimed to explore the sexual function, quality of life, and urinary function of a sample of 26 Brazilian transgender women who have undergone GAS.

Transgender women who were sexually active in our sample were classified without SD. Participants who did not have sexual intercourse in the last four weeks reported a score of 5.63. Although the FSFI evaluates only sexually active women in the previous four weeks, and its highest scores are based on sexual intercourse, it is essential to highlight this difference since, in this population, being or not being sexually active can be affected by factors such as possible surgical complications. Regarding UI, 15% of the sample reported involuntary urine loss. Despite these data, our sample showed good health conditions.

Gender transitioning is increasingly discussed in society. However, there are only a few surgical treatment centers for GD in Brazil and few scientific publications on the topic. To our knowledge, this study is a pioneer in our field in evaluating sexual function and possible alterations in urinary function in Brazilian transgender women who have undergone GAS. The initial hypothesis was that sexual and urinary dysfunction could occur in the medium term, significantly affecting the sexual function, urinary function, and quality of life of transgender women who have undergone surgery. However, the results showed the opposite. Most of the women interviewed were satisfied with GAS. Despite the questionnaire’s results showing that this group of individuals was sexually dysfunctional, transgender women with an active sexual life were classified as sexually functional and received a higher score on the satisfaction domain and a lower one on lubrication. The low score in the lubrication domain is because the neovagina is created from inverted penile skin; that is, it is not mucous tissue and does not have secretory glands. Therefore, the lack of lubrication is an expected limitation of this surgery [20]. However, some participants reported that urethral canal lubrication produced by the remaining Littre glands [21] is sufficient for penetration without discomfort.

Buncamper et al. [22] compared the FSFI scores of a group of transgender women with the score of another study with women whose gender identity matched their phenotypic/biological characteristics (cisgender) and obtained a score similar to the group of women with SD. Our sample is closer to cisgender women with no SD when comparing our results with this study. However, it is essential to note that even women who were sexually dysfunctional and those who were not sexually active, both by choice and by complications of GAS, reported being sexually satisfied. This finding confirms that sexual satisfaction is not only associated with organ function but also with self-confidence, self-image, and body acceptance due to their physical characteristics matching the gender with which they identify. In this regard, Heylens et al. [23] showed that transgender women had a better sexual experience after GAS because they improved their self-image and self-confidence. Furthermore, Lief and Hubschman [24] showed that even patients with decreased sexual function were satisfied due to improved body image.

In the same way, sexual function and satisfaction associated with a better self-image after GAS add to a better quality of life, considering that self-confidence interferes with both the emotional state and interpersonal relationships. Quality of life, in addition to physical health, includes mental, psychological, and social wellness and can be directly associated with the absence of diseases, dysfunctions, and symptoms [25]. Taking the variables evaluated in SF-36, the results revealed an excellent health condition. The highest mean scores were related to functional capacity and emotional aspects. As mentioned previously, these two domains received higher scores due to issues directly associated with self-esteem and social acceptance. Therefore, our findings demonstrated a positive impact on quality of life after GAS, confirming evidence from other studies such as Kuhn et al. [26], van de Grift et al. [27], Salvador et al. [28], and Cardoso et al. [29].

Another important factor in GAS is the impact on the urinary function of transgender women. The literature on urinary changes due to hormonal therapy and GAS is insufficient, and little is known about its long-term impact on the pelvic floor muscles. The studies focused mainly on immediate postoperative urological complications [20,21,22]. In our study, 34.6% of the subjects reported urinary changes, and 19.2% were due to postoperative complications, such as urethral stenosis. The main complaints were difficulty emptying the bladder, forced urination, and a burning sensation when urinating. However, contrary to our expectations, only 15.4% of the sample reported UI and none due to surgery. All individuals said urinary losses started before the surgical procedure but could not tell if they occurred before or after hormonal therapy. Kuhn et al. [26] hypothesized that urinary loss results from hormonal treatment. Their study compared the prostate size of transgender women and cisgender men of the same age. The result was that transgender women chronically exposed to estrogen had a smaller prostate than a group of cisgender men of the same age without hormonal treatment. These data suggest that prostate size can affect urinary continence. The intensity of UI in our sample evaluated by ICIQ-SF ranged from mild to severe. However, none of the transgender women incontinent sought medical attention to treat this urine loss. For this reason, we emphasize the importance of monitoring urinary changes in transgender women from the beginning of hormonal therapy.

This study investigated the perception of transgender women of their sexual and urinary function after GAS, contributing to expanding the knowledge about the psychosexual wellbeing of this population. However, there are some significant limitations to consider. A first limitation reported in this study, as well as in other studies on the same population [30,31], is low adherence to research and dropout of patients after surgery, resulting in small sample sizes and underpowered studies. Since 1998, approximately 160 transgender women have undergone GAS through PROTIG/HCPA, but only 20 have continued to attend support groups. As in our study, Montsrey et al. [32] reported the same difficulties in contacting women after surgery, as they could not be located. When recruiting individuals for our research, most attempts resulted in inexistent or wrong phone numbers. In the absence of clear literature data on this point, we can assume that transgender women after GAS tend to deny and remove the identity which caused them suffering and try to build a new life, i.e., a complete transition to another gender and the possibility of full inclusion in society. Thus, returning to the center where they underwent surgery might be refused to avoid being confronted with their old identity again. Second, physical changes and pelvic floor muscle functionality were not evaluated. For this purpose, it would be necessary to perform a physical examination of these muscles with specific tests performed by specialized physiotherapists. Furthermore, we did not investigate why 15.4% of the sample did not want to penetrate the neovagina. Finally, we had two critical limitations concerning the questionnaires used in this study. In fact, the ICIQ-SF questionnaire is validated only for cisgender men and women and not specifically for transgender women who have altered urinary anatomy and function by GAS, thus leading to a possible evaluation bias. Furthermore, until the beginning of this study, there were no adequate instruments to assess sexual function in transgender women. This specific instrument was only developed and validated in 2020 by Vedovo et al. [33]. Therefore, we chose to use the FSFI despite some known limitations of the instrument, such as assessing sexual activity by referring only to the previous four weeks with the risk of potential bias should sexual activity date with the risk of potential bias if the sexual activity occurred earlier.

## 5. Conclusions

Transgender women in our sample reported a good quality of life and sexual function after GAS. This is due not only to organ functionality but also to the adequacy of self-image. However, since the UI reported by the sample was before GAS, we suggest that future studies explore preoperative data and pre- and post-hormonal therapy to elucidate the causes of UI. In addition, other studies are necessary on the long-term functionality of the pelvic floor muscles and their possible dysfunctions.

## Figures and Tables

**Table 1:** Sample characteristics.

Variables	*n* (%)	Mean (SD)
Age	-	39.9 (10.41)
Weight (kg)	-	74.23 (10.82)
Height (m)	-	1.71 (5.61)
Smoking		
Cigarettes/day	-	1.69 (6.50)
Days after surgery, they resumed smoking	-	40.87 (63.49)
Chronic Diseases		
HIV	5 (19)	-
DM and SAH	1 (3.8)	-
Other unspecified diseases	1 (3.8)	-
Elapsed time of GAS (months)	-	54 (56.40)
Number of genital surgeries	-	1.88 (2.78)
Treatment/postoperative care		
Hygiene	13 (50)	-
Use of a vaginal mold	3 (11.5)	-
Hygiene and use of vaginal mold	10 (38.5)	-
Reasons for dissatisfaction with the result of the GAS		
Large clitoris	1 (3.8)	-
Recurrent fistulas	1 (3.8)	-
No depth in the neovagina	2 (7.7)	-

Notes: Values expressed in absolute frequency (n) and percentages (%).Kilograms (kg). Metro (m). Human immunodeficiency virus (HIV). Diabetes mellitus (DM). Systemic arterial hypertension (SAH).

**Table 2:** Results of the Female Sexual Function Index.

Domain	Mean (SD)	Mean (SD)	*p*-Value	Mean (SD)	*p*-Value
Desire	3.85 (1.42)	4.11 (1.20)	0.198	3.17 (1.82)	0.167
Arousal	3.33(2.14)	4.50 (0.98)	<0.001	0.17 (0.45)	<0.001
Lubrication	2.94 (2.13)	4.03 (1.31)	<0.001	0.00 (0.00)	0.002
Orgasm	3.25 (2.13)	4.34 (1.19)	<0.001	0.29 (0.76)	<0.001
Satisfaction	4.97(1.20)	5.28 (0.59)	<0.020	2.00 (1.70)	*
Pain	3.23(2.46)	4.42 (1.68)	<0.001	0.00 (0.00)	0.003
Total	21.58 (11.47)	26.67 (6.96)	0.023	5.63 (4.73)	*

Test: Wilcoxon–Mann–Whitney. Notes. A total score < 26 can be classified as having sexual dysfunctions * = unable to compute.

**Table 3:** Results of the urinary incontinence questionnaire (ICIQ-SF).

Degree of Incontinence	Score	
	n (%)	
Absence of Urinary Incontinence	22	84.6
Mild Urinary Incontinence (score 1–5)	1	3.8
Moderate Urinary Incontinence (score 6–12)	2	7.7
Severe Urinary Incontinence (score 13–18)	1	3.8
Very Severe Urinary Incontinence (score 19–21)	0	0
Total	26	100.0

Notes. Values are expressed as absolute frequency (n) and relative frequency (%).

**Table 4:** Results of the SF-36 Health Survey.

Domains	Mean	SD	Minimal	Maximum
Physical functioning	93.65	11.88	45.00	100.00
Role—physical	81.73	34.32	0.00	100.00
Bodily pain	73.35	25.40	10.00	100.00
General health	78.31	13.88	52.00	100.00
Vitality	72.88	22.77	15.00	100.00
Social functioning	85.10	24.75	0.00	100.00
Role—emotional	85.90	30.07	0.00	100.00
Mental health	82.77	14.26	52.00	100.00

## Data Availability

The data presented in this study are available on request from the corresponding author. The data are not publicly available to protect participants’ privacy.

## Supplementary Materials

The following supporting information can be downloaded at: https://www.mdpi.com/article/10.3390/ijerph192315773/s1, Table S1: STROBE checklist.

## References

[1] American Psychiatric Association (2013). Diagnostic and Statistical Manual of Mental Disorder.

[2] Lobato M.I., Koff W.J., Manenti C., da Fonseca Seger D., Salvador J., da Graca Borges Fortes M., Petry A.R., Silveira E., Henriques A.A. (2006). Follow-up of sex reassignment surgery in transsexuals: A Brazilian cohort. Arch. Sex. Behav..

[3] Oles N., Darrach H., Landford W., Garza M., Twose C., Park C.S., Tran P., Schechter L.S., Lau B., Coon D. (2022). Gender Affirming Surgery: A Comprehensive, Systematic Review of All Peer-reviewed Literature and Methods of Assessing Patient-centered Outcomes (Part 2: Genital Reconstruction). Ann. Surg..

[4] Javier C., Crimston C.R., Barlow F.K. (2022). Surgical satisfaction and quality of life outcomes reported by transgender men and women at least one year post gender-affirming surgery: A systematic literature review. Int. J. Transgend. Health.

[5] Safa B., Lin W.C., Salim A.M., Deschamps-Braly J.C., Poh M.M. (2019). Current Concepts in Feminizing Gender Surgery. Plast. Reconstr. Surg..

[6] Brasil A.P.A., Abdo C.H.N. (2016). Transtornos sexuais dolorosos femininos. Diagn. Tratamento.

[7] Caruso S., Bandiera S., Cavallaro A., Cianci S., Vitale S.G., Rugolo S. (2010). Quality of life and sexual changes after double transobturator tension-free approach to treat severe cystocele. Eur. J. Obstet. Gynecol. Reprod. Biol..

[8] Khajehei M., Doherty M., Tilley P.J. (2015). An update on sexual function and dysfunction in women. Arch. Womens Ment. Health.

[9] von Elm E., Altman D.G., Egger M., Pocock S.J., Gotzsche P.C., Vandenbroucke J.P., Initiative S. (2014). The Strengthening the Reporting of Observational Studies in Epidemiology (STROBE) Statement: Guidelines for reporting observational studies. Int. J. Surg..

[10] Brazilian Ministry of Health Portaria n°2803: Redefine e amplia o Processo Transexualizador no Sistema Único de Saúde (SUS). https://bvsms.saude.gov.br/bvs/saudelegis/gm/2013/prt2803_19_11_2013.html.

[11] Brazilian Ministry of Health Portaria n°170. https://bvsms.saude.gov.br/bvs/saudelegis/sas/2008/prt0170_20_03_2008.html.

[12] Brazilian Ministry of Health Portaria n°457. https://bvsms.saude.gov.br/bvs/saudelegis/sas/2008/prt0457_19_08_2008.html.

[13] World Health Organization ICD-11: International classification of diseases (11th revision). https://icd.who.int/.

[14] Thiel Rdo R., Dambros M., Palma P.C., Thiel M., Riccetto C.L., Ramos Mde F. (2008). Translation into Portuguese, cross-national adaptation and validation of the Female Sexual Function Index. Rev. Bras. Ginecol. Obstet..

[15] Ciconelli R., Ferraz M., Santos W., Meinao I., Quaresma M. (1999). Brazilian-Portuguese version of the SF-36. A reliable and valid quality of life outcome measure. Rev. Bras. Reumatol..

[16] Tamanini J.T., Dambros M., D’Ancona C.A., Palma P.C., Rodrigues Netto N. (2004). Validation of the “International Consultation on Incontinence Questionnaire—Short Form” (ICIQ-SF) for Portuguese. Rev. Saude Publica.

[17] Rosen R., Brown C., Heiman J., Leiblum S., Meston C., Shabsigh R., Ferguson D., D’Agostino R. (2000). The Female Sexual Function Index (FSFI): A multidimensional self-report instrument for the assessment of female sexual function. J. Sex Marital Ther..

[18] Wiegel M., Meston C., Rosen R. (2005). The female sexual function index (FSFI): Cross-validation and development of clinical cut-off scores. J. Sex Marital Ther..

[19] Riccetto C., Palma P., Herrmamm V., Dambros M., Thiel M., Tamanini J.T.N., Netto N.R. (2005). 1315: Is There Correlation between Urodynamic Findings and International Consultation on Incontinence Questionnaire—Short form (ICIQ-SF) Score?. J. Urol..

[20] Buncamper M.E., van der Sluis W.B., van der Pas R.S.D., Ozer M., Smit J.M., Witte B.I., Bouman M.B., Mullender M.G. (2016). Surgical Outcome after Penile Inversion Vaginoplasty: A Retrospective Study of 475 Transgender Women. Plast Reconstr. Surg..

[21] Papadopulos N.A., Lelle J.D., Zavlin D., Herschbach P., Henrich G., Kovacs L., Ehrenberger B., Kluger A.K., Machens H.G., Schaff J. (2017). Quality of Life and Patient Satisfaction Following Male-to-Female Sex Reassignment Surgery. J. Sex. Med..

[22] Buncamper M.E., Honselaar J.S., Bouman M.B., Ozer M., Kreukels B.P., Mullender M.G. (2015). Aesthetic and Functional Outcomes of Neovaginoplasty Using Penile Skin in Male-to-Female Transsexuals. J. Sex. Med..

[23] Heylens G., Verroken C., De Cock S., T’Sjoen G., De Cuypere G. (2014). Effects of different steps in gender reassignment therapy on psychopathology: A prospective study of persons with a gender identity disorder. J. Sex. Med..

[24] Lief H.I., Hubschman L. (1993). Orgasm in the postoperative transsexual. Arch. Sex. Behav..

[25] Castellano E., Crespi C., Dell’Aquila C., Rosato R., Catalano C., Mineccia V., Motta G., Botto E., Manieri C. (2015). Quality of life and hormones after sex reassignment surgery. J. Endocrinol. Invest..

[26] Kuhn A., Bodmer C., Stadlmayr W., Kuhn P., Mueller M.D., Birkhauser M. (2009). Quality of life 15 years after sex reassignment surgery for transsexualism. Fertil. Steril..

[27] van de Grift T.C., Elaut E., Cerwenka S.C., Cohen-Kettenis P.T., Kreukels B.P.C. (2018). Surgical Satisfaction, Quality of Life, and Their Association After Gender-Affirming Surgery: A Follow-up Study. J. Sex. Marital Ther..

[28] Salvador J., Massuda R., Andreazza T., Koff W.J., Silveira E., Kreische F., de Souza L., de Oliveira M.H., Rosito T., Fernandes B.S. (2012). Minimum 2-year follow up of sex reassignment surgery in Brazilian male-to-female transsexuals. Psychiatry Clin. Neurosci..

[29] Cardoso da Silva D., Schwarz K., Fontanari A.M., Costa A.B., Massuda R., Henriques A.A., Salvador J., Silveira E., Elias Rosito T., Lobato M.I. (2016). WHOQOL-100 Before and After Sex Reassignment Surgery in Brazilian Male-to-Female Transsexual Individuals. J. Sex. Med..

[30] Kuhn A., Hiltebrand R., Birkhauser M. (2007). Do transsexuals have micturition disorders?. Eur. J. Obstet. Gynecol. Reprod. Biol..

[31] Lawrence A.A. (2003). Factors associated with satisfaction or regret following male-to-female sex reassignment surgery. Arch. Sex. Behav..

[32] Monstrey S., Hoebeke P., Dhont M., Cuypere G.D., Rubens R., Moerman M., Hamdi M., Landuyt K.V., Blondeel P. (2020). Surgical Therapy in Transsexual Patients: A Multidisciplinary Approach. Acta Chir. Belg..

[33] Vedovo F., Di Blas L., Perin C., Pavan N., Zatta M., Bucci S., Morelli G., Cocci A., Delle Rose A., Caroassai Grisanti S. (2020). Operated Male-to-Female Sexual Function Index: Validity of the First Questionnaire Developed to Assess Sexual Function after Male-to-Female Gender Affirming Surgery. J. Urol..

